# Association between religiosity and orthorexia nervosa with the mediating role of self-esteem among a sample of the Lebanese population – short communication

**DOI:** 10.1186/s40337-022-00672-0

**Published:** 2022-10-24

**Authors:** Michel Sfeir, Diana Malaeb, Sahar Obeid, Souheil Hallit

**Affiliations:** 1 Institute of Psychology, Faculty of Social and Political Sciences, University of Lausanne, Lausanne, Switzerland; 2 College of Pharmacy, Gulf Medical University, Ajman, UAE; 3 School of Pharmacy, Lebanese International University, Beirut, Lebanon; 4 Social and Education Sciences Department, School of Arts and Sciences, Lebanese American University, Jbeil, Lebanon; 5 School of Medicine and Medical Sciences, Holy Spirit University of Kaslik, P.O. Box 446, Jounieh, Lebanon; 6 Research Department, Psychiatric Hospital of the Cross, Jal Eddib, Lebanon

**Keywords:** Orthorexia nervosa, Self-esteem, Religiosity, Lebanon

## Abstract

**Background:**

Orthorexia Nervosa is not yet classified as an eating disorder albeit it can be found in different populations. This condition can be characterized by a preoccupation with the quality of food, accompanied by obsessive thoughts regarding eating behaviors, leading to malnutrition. Previous associations have been reported between high levels of eating disorders and lower levels of self-esteem; where individuals have low self-esteem due to the pressured felt to fit the norms of society in beauty standards. The aim of the present study was to evaluate the relationship between religiosity and orthorexia nervosa via either trait or state self-esteem.

**Methods:**

This study was conducted between September 2021 and February 2022 and included 428 participants from all Lebanese governorates. The Teruel Orthorexia Nervosa scale was used to measure orthorexia nervosa. The following scales state self-esteem and religiosity were used to measure self-esteem.

**Results:**

Sociodemographic characteristics (age, gender, marital status, household crowding index, body mass index and education) were entered in the mediation model as confounding variables. Higher religiosity was significantly associated with higher state self-esteem (Beta = 0.07), while higher state self-esteem was significantly associated with lower identification of those that exhibited ON tendencies or symptoms (Beta= -0.11).

**Conclusion:**

A high state self-esteem was correlated with a lower level of orthorexia nervosa. Higher religiosity was shown to be associated with higher self-esteem, which in turn was associated with a decrease in the scores of orthorexia nervosa.

## Introduction

Self-esteem, described by the positive or negative value people attribute to themselves, is considered to be a basic need for humans [1, 2]. The construct of self-esteem, referred to as a trait, can be considered stable and global, includes the personal judgement of one’s value or worth [3]. Crouch & Straub (1983) have argued that a person’s trait self-esteem is developed and relatively unchangeable in adulthood, and hence this self-esteem trait is relatively stable [4]. The global aspect of self-esteem can be explained by the presence of both positive and negative feelings one might have towards themselves as measured by the Rosenberg Self-Esteem Scale (RSS) [5]. Global self-esteem, as explained by previous research, can include several domains such as: social, cognitive and physical self-esteems [6, 7]. Self-esteem is a flexible construct, it goes through fluctuations or small changes, where Heatherton and Polivy in 1991 distinguished it as a state of self-esteem [8]. Self-esteem state fluctuates depending on people approval by others [9] and consists of how individuals evaluate their self-worth. Unlike general or trait self-esteem which could be considered to be stable over time, Crouch and Straub suggested that a person’s trait self-esteem can be established by adulthood [4, 10]. On the other hand, Butler and colleagues referred to “self-esteem lability” to explain the changes in the state self-esteem in different contexts or situations [11].

For instance, the self-esteem state can refer to this change in self-esteem when someone is sick or unemployed [4]. This close relationship between trait self-esteem and state self-esteem is reduced when something might threaten the ego [8]. The reason for distinguishing between these two forms in the current study was to evaluate the difference between the trait self-esteem and the state self-esteem in the midst of the COVID-19 pandemic and the economic crisis happening in Lebanon. We hypothesized that the state self-esteem would be more significantly associated with Orthorexia Nervosa (ON). Moreover, previous research has found no significant association between trait self-esteem and ON while using the RSS which evaluated the self-esteem trait [5, 12, 13]. In-addition, there is absence in previous research that evaluated state self-esteem with ON or any form of self-esteem as a mediator between ON and religiosity.

Self-esteem can also be correlated with self-image, where a negative self-image of oneself was correlated with a negative self-esteem [14]. In fact, self-image is usually shaped by primary attachment styles or persons and it plays an important role in the interactions with others [15]. People with eating disorders scored more negative scores of self-images than controls [16, 17].

In 2019, a study showed that a higher score of eating disorder was associated with a lower self-esteem [18]. Previous literature showed that females with eating disorders were associated with a lower self-esteem where individuals may feel the pressure to fit society’s norms of beauty ideals [19, 20, 21]. A higher self-esteem was positively associated with a higher general wellbeing [22], whereas a low self-esteem was linked with more substance abuse/use as well as eating disorders [23].

Recent literature links mental health as well as other domains to religion [24, 25]. A cross-sectional study argued that religion has a great effect on human health and could provide people with a positive mental state [26]. In terms of self-esteem, previous research showed that higher levels of religiosity were associated with higher self-esteem score [27, 28]. Religious beliefs were positively associated with a higher self-esteem as well as better mental health [29].

Just like self-esteem, eating disorders were associated with spirituality, where strong religious beliefs were correlated with lower concerns about the body as well as disordered eating [30]. In a study conducted on Dutch health professionals, a 50-year-old female stated that the insecurities are emerging and one of the factors was that religion was gone and people were looking for a way to deal with the insecurities and fall back on, like ON or even Anorexia Nervosa [31]. To this date, there was no study that evaluated the association of religiosity with Orthorexia Nervosa. Religiosity can refer to several aspects associated with religious beliefs as well as involvements [32]. The beliefs would refer to a reverence for a deity and the involved would be engaging in activities or services related to this faith [33]. Previous authors have stated that religiosity also referred to as religiousness can be defined as the “strength of one’s connection to or conviction for their religion” [34]. Religion in this case is related to the practices, beliefs and behaviors associated with the idea of the “transcendent” that can be practiced individually or within a community [35].

ON, still not classified in the Diagnostic and Statistical Manual of Mental Health Disorder 5 (DSM-5), nor in the International Classification of Diseases, Eleventh Revision (ICD-11), can be explained as beliefs and behaviors that are obsessive and compulsive - respectively - in regards to eating behaviors [36]. The main concern of people with ON would be the healthy quality of the diet rather than the quantity, as in making sure of the presence of nutrients, preservatives or the use of pesticides, etc. [36–38]. This unhealthy obsession with healthy eating and strict dieting might lead to negative nutritional repercussions [36].

ON tendencies, according to Renee McGregor, are a self-development obsession rather than only striving for a perfect weight. The author describes the origin of ON tendencies to when people challenge themselves by pushing their limits in order to gain self-esteem [39]. In a previous research, bloggers identified with ON tendencies expressed that they felt the need to be in control and to reach perfection which maintained their disordered eating. This “punishing drive for perfection and control” as identified by the authors, pushed the participants to obsessively be in control of their diet and exercise by implementing strict rules [40]. Some participants shared that they became judgmental while using downward comparisons in regards of other people’s health life choices which brought them a sense of superiority. People with a low self-esteem might be particularly interested in social comparison which might increase their satisfaction [41, 42]. Moreover, self-esteem was previously shown to be a predictor for ON tendencies where higher scores on the ON scale were correlated with lower levels of self-esteem [43].

People with ON may also experience emotional distress as well as social and educational impairments on both physical and physiological levels [44, 45]. In addition, they may experience a high level of frustration when they are not satisfied with their food practices and a feeling of guilt when they do not follow their diet or transgress it [46]. ON was also associated with disordered eating attitudes [47], such as a severe restriction over food, unhealthy behaviors as well as diet habits to lose or maintain weight [48].

The association of ON and gender seemed to be controversial across several studies. Two studies found that ON tendencies were more prevalent in women than men [49, 50], whereas two other studies found the opposite [37, 51]. Few more studies found no significant correlations between the two [13, 52, 53]. A previous study found a significant association between ON and marital status in a Lebanese sample whereas another cross-cultural study conducted between Lebanon and Germany found no association between the two [54, 55]. Moreover, Bona and colleagues (2021) found that ON tendencies were more present in younger participants [43].No previous significant correlation was found between ON and other sociodemographic data such as education and household crowding index.

**Fig. 1:**
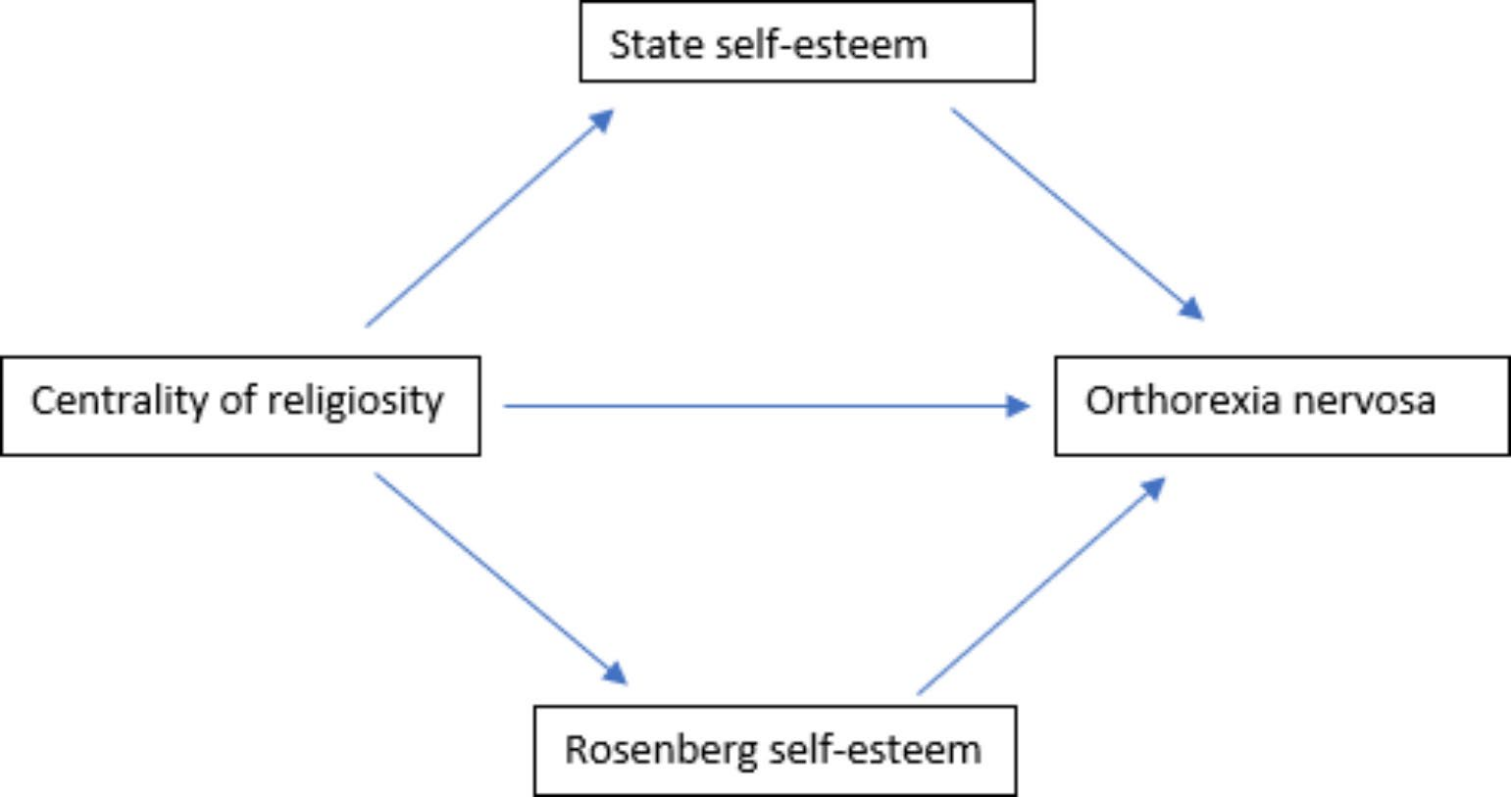
Conceptual framework of the association between religiosity and ON with the mediating role of self-esteem [29, 43, 56, 57]

The following conceptual model **(**Fig. 1**)** represents the association between religiosity and ON with the mediating role of self-esteem. When an individual has a peaceful approach and has a higher level of religiosity, the less they want to validate their looks or rely on an uncomfortable wish to look healthy. The greater the religious awareness is, the lower the ON would be. Self-esteem can be associated with the negative feelings and the worries to appear in a healthy bod, and this association could be valid through the mediator role of spiritual awareness [43]. A higher level of intrinsic religion can play a protective factor against eating disorders [56]. As religious beliefs can help increase self-esteem [29], some evidence suggests that an enhanced self-esteem can play a protective factor against eating disorders [57]. With Lebanon being a religious country, religiosity was extensively studied in association of several mental health and psychology concepts such as suicidal risk, depression, addiction [58–60], the current study aimed to evaluate the association of religiosity with ON with a mediating role of self-esteem.

Lebanon is considered to be a diverse country as per religions where we find Islam and Christianity as well as other religions (https://www.state.gov/reports/2019-report-on-international-religious-freedom/lebanon/ accessed on 6 March, 2022). Most Lebanese would consider God as being full of power and the source of miracles, and religion plays a vital role in their life [61]. Previous research has shown the importance of religion in people’s life and illnesses where participants felt that their illness had a purpose. The authors of this previous study also recommended the integration of spiritual care to make sure patients have a better quality of life [62]. In a sample of 519 Lebanese individuals, the prevalence of ON tendencies (using the Dusseldorf Orthorexia Scale) was found to be present in 8.4% of the entire sample and 17.5% of the sample was at risk of developing ON [54]. In another study conducted on Lebanese individuals, the authors have highlighted the need for social awareness and behavioral interventions to treat ON [63]. The importance of this social awareness would be for people to be more vocal about eating disorders and especially about ON in order to be able to plan interventions and treatment plans accordingly.

With this being said and the scarce literature about ON in the Arab countries, the goal of the present study was to assess the association of higher religiosity with lower ON in a sample of the Lebanese population with the mediating role of self-esteem. We expected that self-esteem would mediate the association between religiosity and ON. Moreover, we used state self-esteem in this current study to study the fluctuation of self-esteem in the moment in which participants filled the questionnaire. This would give us a better idea on how they would feel at the time.

## Methods

### Study design

This study was carried out from September 2021 till February 2022 during quarantine restrictions imposed by COVID-19 pandemic. The data was collected online through a questionnaire designed on Google Forms where the questionnaire was shared online on several platforms (WhatsApp, LinkedIn, and Instagram) in order to reach the target number. The recruitment of this study was a general community sample that was collected from all over Lebanon using the snowball technique. A small description of the goal of the study and the criteria to participate (older than 18 and being a Lebanese citizen) were shared as well. Non-validated scales were translated from English to Arabic and then a back-translation was done to ensure a standardized translation. An Arabic-speaking psychologist first translated the scales from English to Arabic and sent it to an English-speaking psychologist who was not familiar with the scales who did the back-translation to English.

### Minimal sample size calculation

A minimal sample of 410 was deemed necessary using the formula suggested by Fritz and MacKinnon [64] to estimate the sample size: $n=\frac{L}{f2}+k+1$, where f=0.14 for small effect size, L=7.85 for an α error of 5% and power β = 80%, and k=8 variables to be entered in the model.

### Questionnaire

The questionnaire was designed in Arabic. The first part of the questionnaire targeted the sociodemographic data of the participants (age, sex, marital status and educational level). Body Mass Index (BMI) was calculated from self-reported height and weight. Household crowding index, reflecting the socioeconomic status, consists of the number of persons divided by the number of rooms in the house [65].

#### Teruel Orthorexia Scale (TOS)

The TOS is composed or 17 items that includes two aspects of orthorexia. The two aspects are Healthy Orthorexia and Orthorexia Nervosa and each one has respectively 9 and 8 items [66], (in this study, Cronbach’s alpha for ON = 0.86 and 0.88 for HeOr). The scale was validated in Lebanon [67] and was previously used among Lebanese samples with a good reliability score both subscales [68]. In this paper, the TOS ON subscale will be used.

#### State Self-Esteem Scale (SSES)

The SSES [8]( evaluated the fluctuations of self-esteem and consists of 20 items in which there are 13 reversed items. Items are rated from a scale of 1 to 5 (1 = Not At all, 5 = extremely), (in this study, Cronbach’s alpha = 0.91). Higher scores would indicate higher State Self-Esteem [8]. This scale was validated by Heatherton & Polivy (1991) in participants aged from 23 to 57 years.

#### Rosenberg Self-Esteem Scale (RSS)

The RSS [5] was used to evaluate trait self-esteem. It is composed of 10 items, in which 5 items are reversed. This scale is scored as a Likert scale, with a 4-point response from Strongly Disagree to Strongly Agree (in this study, Cronbach’s alpha = 0.66). This scale measured the global or trait self-esteem [69]. RSS was previously validated in research that included more than 15,000 adults’ participants across several countries [70].

#### Centrality of Religiosity Scales (CRS)

The CRS [71] assessed the importance and centrality of religiosity in individuals. It consists of 15 items, 12 of the items ranging from 1 = Never to 5 = Very much, and the 3 others consisted of rating of 8-points, from Several times a day to Never (in this study, Cronbach’s alpha = 0.94). Adequate psychometric qualities were reported by a validation among Brazilian adults [72].

### Statistical analysis

There was no missing data since all questions were required in the Google form link. The TOS orthorexia nervosa score was considered normally distributed (skewness and kurtosis values between − 2 and + 2) [73] .The SPSS software v.22 was used to conduct the bivariate analysis. The Student t test was used to compare two means, whereas the Pearson correlation test was used to correlate two continuous scores. The PROCESS SPSS Macro version 3.4, model four [74] was used to calculate all pathways (Pathway A from the independent variable to the mediator, Pathway B from the mediator to the dependent variable and Pathway C from the independent to the dependent variable). Pathway AB calculated the indirect effect; the latter was deemed significant when the macro generated bias-corrected bootstrapped 95% confidence intervals (CI) did not pass by zero [74]. The covariates that were included in the mediation model were age, gender, education level, BMI, household crowding index.

## Results

### Sociodemographic characteristics of the participants

A total of 428 participants was included in this study, with a mean age of 23.57 ± 7.38 years (min = 18; max = 57), 283 (66.1%) females, 383 (89.5%) single, and 406 (94.9%) with a university level of education. In addition, the mean BMI was 23.43 ± 4.41 kg/m2, whereas that of the household crowding index was 0.96 ± 0.47 persons/room **(**Table 1**).**

**Table 1:** Sociodemographic characteristics of the participants

Variable	N (%)
Gender	
Male	145 (33.9%)
Female	283 (66.1%)
Marital status	
Single	383 (89.5%)
Married	45 (10.5%)
Education	
Secondary or less	22 (5.1%)
University	406 (94.9%)
	**Mean ± SD**
Age (in years)	23.57 ± 7.38
Body Mass Index (kg/m^2^)	23.43 ± 4.41
Household crowding index (persons/room)	0.96 ± 0.47
TOS orthorexia nervosa	12.67 ± 4.58
Rosenberg self-esteem	15.82 ± 1.74
State self-esteem	70.16 ± 12.69
Central religiosity	81.28 ± 21.57

### Bivariate analysis

The bivariate analysis results are displayed in Table 2. Higher state self-esteem (r = − 0.27) was significantly associated with lower ON tendencies or symptoms, whereas higher body mass index (r = 0.15) was significantly associated with higher ON tendencies or symptoms. No significant difference was seen between males and females (12.88 vs. 12.55; p = 0.484), single and married participants (12.61 vs. 13.11; p = 0.491) and those with a secondary level of education or less vs. university level (12.36 vs. 12.68; p = 0.751) in terms of orthorexia nervosa.

**Table 2:** Correlation of continuous variables with orthorexia nervosa

Variable	TOS ON	Rosenberg self-esteem	State self-esteem	Central religiosity	Age	Body Mass Index	Household crowding index
TOS ON	1						
Rosenberg self-esteem	− 0.06	1					
State self-esteem	− 0.27***	0.35***	1				
Central religiosity	0.06	0.08	0.13**	1			
Age	0.02	0.07	0.11*	0.08	1		
Body Mass Index	0.15**	− 0.05	− 0.03	− 0.02	0.19***	1	
Household crowding index	0.04	− 0.01	0.01	0.12*	-0.16**	− 0.05	1

TOS ON = TOS orthorexia nervosa subscale; ***p < 0.01; **p < 0.01; *p < 0.05

### Indirect effect of self-esteem

**Fig. 2:**
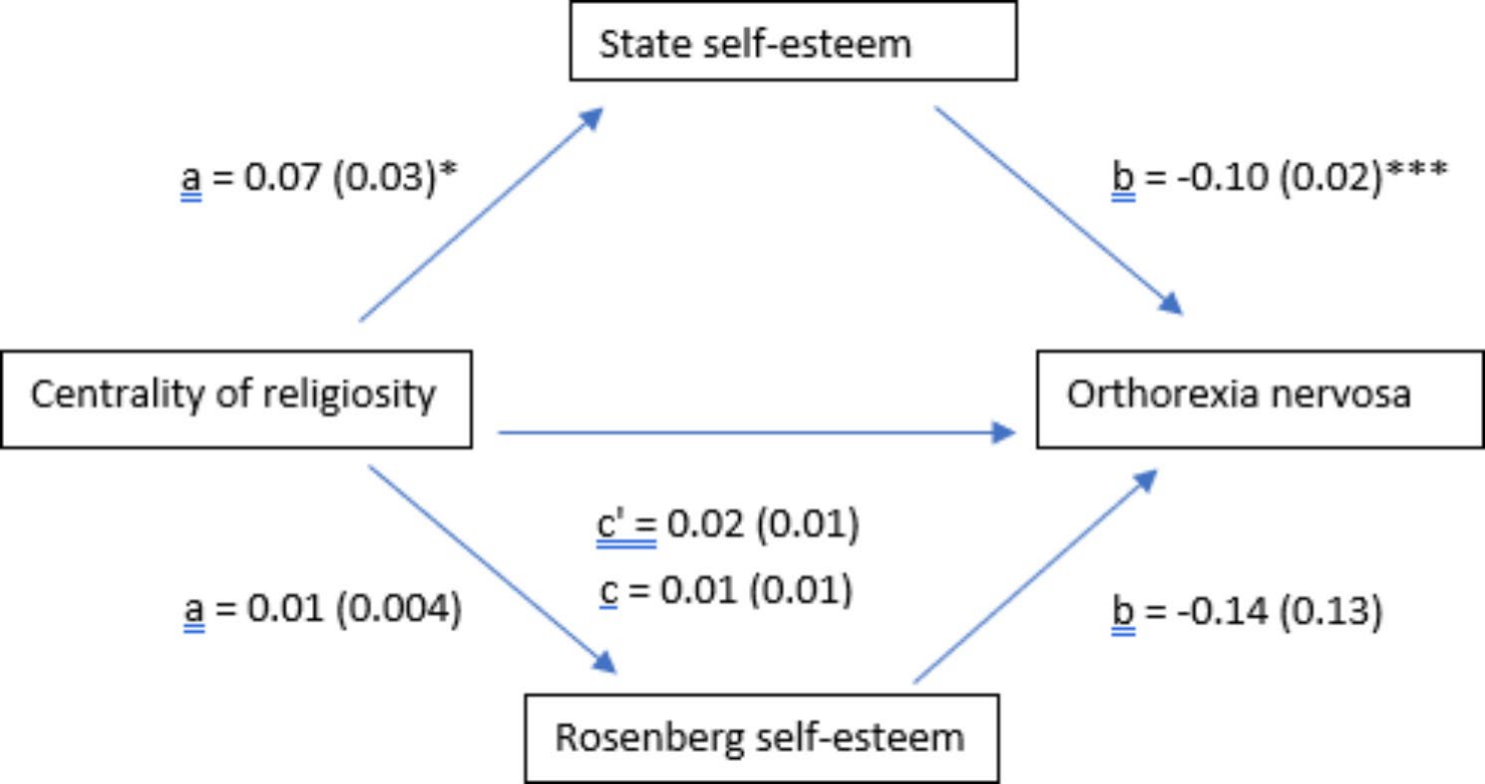
(a) Relation between religiosity and state self-esteem; (b) Relation between state self-esteem and orthorexia nervosa; (c) total effect of religiosity and orthorexia nervosa; (c’) direct effect of religiosity on orthorexia nervosa. Numbers are displayed as regression coefficients (standard error). *p < 0.05; ***p < 0.001

The results of the mediation analysis were adjusted over all sociodemographic characteristics. State self-esteem [95% BCa of the indirect effect: -0.01; -0.001], but not trait self-esteem [95% BCa of the indirect effect: -0.003; 0.001], had a significant indirect effect between religiosity and identification of those that exhibited ON tendencies or symptoms. Higher religiosity was significantly associated with higher state self-esteem (Beta = 0.07), while higher state self-esteem was significantly associated with lower identification of those that exhibited ON tendencies or symptoms (Beta= -0.10) (Fig. 2).

## Discussion

The aim of the current study was to investigate the association between religiosity and ON tendencies with the mediating role of self-esteem. Our results showed that higher state self-esteem was associated with lower ON, which aligns with previous results showing self-esteem as a predictor for ON [43]. Bona and colleagues (2021) tried to explain the relationship between self-esteem and ON by a fear coming from the pressures placed by society. It would be accepted on a social level to have strict and controlled diets because this would indicate having a healthy consciousness [75]. Moreover, a low score of self-esteem was also associated with eating disorders [76]. Previous studies have found no significant association between trait self-esteem scores and ON tendencies [12] and other studies have found that ON tendencies were positively associated with trait self-esteem [36]. This could be due to the assessment of trait self-esteem as in the current study where only state self-esteem was significantly negatively associated with ON tendencies. Some authors have found that having a high score of self-esteem reduced the tendency to healthy eating obsession making low self-esteem a susceptible predisposing factor for ON tendencies [77].

The results of our study found that state-self-esteem had a significant indirect effect between religiosity and ON. We predicted in our hypothesis a mediating role of self-esteem between religiosity and ON. Religiosity in this case refers to both religious beliefs and engagement in religious activities affiliated to one’s religion and can also be referred to as religiousness. Bona and colleagues, found that the higher the spiritual awareness was, the less likely the participants were found to have symptoms of orthorexia. People who have a high score on spirituality would generally not be concerned about other people’s opinions [78]. This would mean that with this spiritual approach, people’s self-esteem would not be low as they would not engage in restrictive diets or preoccupy themselves with healthy eating habits, which aligns with our theoretical framework and our initial hypothesis. Self-esteem could play a protective role against adverse situations and help reduce the impact of negative events [79, 80]. Almost 70% of reports, showed that religious individuals have higher levels of self-esteem [81] which can be interpreted by the fact that positive image of God and attending church duties, predicted higher levels of self-esteem among young people [82, 83]. This aligns with the results of this current study showing the association of religiosity with self-esteem. These results also align with previous research in which religious comfort and more religiosity were positively correlated with higher self-esteem [81, 84]. People might use self-esteem as a way to help themselves in adverse situations or dangers coming from the environment [85]. As no previous study evaluated the association of state self-esteem with religiosity, we assume that in the midst of the current situation in Lebanon, the fluctuation of people’s state self-esteem was significantly correlated with religiosity as people might be using religiosity as a way to cope with the daily stress. Another interpretation of the significant association of state self-esteem as mediator might be due to the many challenges faced by the Lebanese community on a daily basis. Alongside with the economic inflation in Lebanon, COVID-19, social insecurity, political instability as well as the increase in unemployment could be possibly associated with this fluctuation of state self-esteem. As trait self-esteem is considered to be a stable overtime it might not change immediately after challenging situations. Previous research has indeed found significant associations between the above-mentioned challenges in Lebanon with mental health aspects and mental health disorders [86–89]. This could, in some way, explain how state self-esteem – that can be changeable from one context to another – mediated the association of religiosity and ON.

### Limitations

Information bias is plausible since answers might not always be correct. A selection bias is possible because of the snowball technique used during the data collection; therefore, our results are not generalizable. More variables known to be associated with ON were not considered, predisposing us to a residual confounding bas.

## Conclusion

Our study showed that state self-esteem had an indirect effect on religiosity and ON in our sample. Religiosity should be given more focus in the clinical practice, especially in Lebanon as it is considered as a religious country. This approach could be used by clinicians in their treatment plans in order to help some of their clients better.

## Declarations

### Availability of supporting data

Data is not available publicly; it is available upon a logical and valid request to the corresponding author.

## Abbreviations

ON: Orthorexia nervosa
TOS: Teruel Orthorexia Scale
SSES: State Self-Esteem Scale
RSS: Rosenberg Self-Esteem Scale
CRS: Centrality of Religiosity Scale
